# Stochastic Particle Creation: From the Dynamical Casimir Effect to Cosmology

**DOI:** 10.3390/e25010151

**Published:** 2023-01-11

**Authors:** Matías Mantiñan, Francisco D. Mazzitelli, Leonardo G. Trombetta

**Affiliations:** 1 Department of Physics, University of Chicago, Chicago, IL 60637, USA; 2 Centro Atómico Bariloche and Instituto Balseiro, Comisión Nacional de Energía Atómica, Bariloche R8402AGP, Argentina; 3 CEICO, Institute of Physics of the Czech Academy of Sciences, Na Slovance 1999/2, 18221 Prague, Czech Republic

**Keywords:** dynamical Casimir effect, stochastic particle creation, cosmology

## Abstract

We study a stochastic version of the dynamical Casimir effect, computing the particle creation inside a cavity produced by a random motion of one of its walls. We first present a calculation perturbative in the amplitude of the motion. We compare the stochastic particle creation with the deterministic counterpart. Then, we go beyond the perturbative evaluation using a stochastic version of the multiple scale analysis, that takes into account stochastic parametric resonance. We stress the relevance of the coupling between the different modes induced by the stochastic motion. In the single-mode approximation, the equations are formally analogous to those that describe the stochastic particle creation in a cosmological context, that we rederive using multiple scale analysis.

## 1. Introduction

Quantum systems under the influence of time-dependent external conditions have attracted a lot of interest in the last decades. One of the interesting phenomena that arises in this context is particle creation induced by time-dependent external backgrounds on quantum fields. The time dependence can be produced by time-dependent properties of the media in which the field propagates: by an external (e.g., electromagnetic or gravitational) field, or by time-dependent boundary conditions. Concrete examples are the so-called dynamical Casimir effect (DCE) [1,2,3,4,5], in which photons are created by moving mirrors or by time-dependent electromagnetic properties, and gravitational particle creation for quantum field theories in curved spaces [6,7,8,9,10]. The most studied situations in the last context are cosmological particle creation and Hawking radiation.

Most previous works on these subjects consider deterministic time-dependent external conditions. In this paper we are interested in the case of stochastic external conditions.

In the cosmological context, to our knowledge, Ref. [11] addressed the problem for the first time, considering a quantum field in the presence of a background metric with a random component. In more recent works [12,13], the stochasticity comes from the interaction of the quantum field with different environments. Technically, the dynamics of the system are analyzed using a correspondence to one-dimensional quantum mechanical problems with random impurities (more precisely, electrical resistance in wires with impurities). The role of noise in the early Universe has been discussed in [14].

Regarding previous works in the context of DCE, in Ref. [15], the authors considered a single harmonic oscillator and assumed that the time-dependent frequency consists of a sequence of barriers. The number of photons is evaluated assuming stochastic amplitudes and stochastic separation between barriers. This separation turns out to be more crucial than the amplitude fluctuations. Contact is made with the problem of impurities in one-dimensional chains, as in the cosmological approach described above. In Ref. [16], the problem is treated at the quantum mechanical level for both the mirror and a single electromagnetic mode. On the other hand, in Ref. [17], the authors considered the DCE produced by a moving mirror that oscillates with a frequency given by $\Omega =2\omega_{0}+K$, where $\omega_{0}$ is the lowest eigenfrequency of the modes in the cavity and *K* is a stochastic detuning.

Different approaches have been used to tackle the problem of the deterministic DCE. In 1+1 dimensions, the conformal invariance allows for a simple solution, as described in the seminal paper by Moore [1]. The problem can also be addressed using the so-called instantaneous basis approach, in which the field is expanded in modes that satisfy the boundary conditions at each time [18]. This method can be extended to 3+1 dimensions [19], and also to the case of the full electromagnetic field [20]. The equations for the modes become those of a set of coupled harmonic oscillators with time-dependent frequencies and couplings. To solve these dynamical equations, one can work perturbatively in the amplitude of the motion of the walls. Alternatively, for oscillatory motion, one can go beyond perturbation theory using the so-called multiple scale analysis (MSA) [21]. This method produces a resumation of the secular terms that appear in the perturbative calculations, and gives an exponential number of created particles due to parametric resonance.

In this paper, we discuss the phenomenon of stochastic particle creation (SPC). For the DCE, we go beyond the single-mode approximation discussed in previous works, computing the SPC first in a perturbative approach, and then using a stochastic version of the MSA. Due to parametric stochastic resonance, the number of created particles grows exponentially, although with a slower rate than in the deterministic case.

For the case of a single mode, the dynamical equation that describes the DCE is equivalent to the equation of each mode of a scalar field in a cosmological context: a harmonic oscillator with stochastic frequency. We compare our results, based on the MSA, with those obtained in previous works.

The paper is organized as follows. In the next section we review the standard approach to the DCE, based on the instantaneous basis approach for a quantum scalar field. In Section 3, we solve the equations for the modes assuming a stochastic motion of the wall. We compute the Bogoliubov coefficients perturbatively in the amplitude of the (random) motion, that depends on the correlation function of the noise. We discuss whether it is possible or not to neglect the intermode couplings. In Section 4, we describe a nonperturbative calculation for a single mode, based on a stochastic generalization of the MSA method, as described originally in Ref. [22]. We present a simpler version of the calculation, to highlight the relation between the stochastic and the deterministic MSA. In Section 5, we generalize the stochastic MSA for a set of coupled oscillators, and discuss its relevance in the DCE. In Section 6, we use the results of Section 4 to analyze SPC in cosmology. Section 7 contains the conclusions of our work.

## 2. Quantum Scalar Field in a Cavity

In this section, we review the quantization of a scalar field in a cavity, using the instantaneous basis formalism. We follow closely Ref. [19], with slight changes of notation.

We consider a scalar field in a rectangular cavity formed by perfect mirrors, with dimensions $L_{x},L_{y},$ and $L_{z}$. The mirrors placed at $x=L_{x}$ and $y=L_{y}$ are at rest, while the other follows a prescribed trajectory $z=L_{z}\left(t\right)$. We will assume that the motion starts at t=0 and finishes at t=T, when the mirror returns to its original position.

The scalar field ϕ(x,t) satisfies the wave equation □ϕ=0 in 3+1 dimensions, and Dirichlet boundary conditions ${\phi |}_{\mathrm{walls}}=0$ for all times. As usual, we expand the field in terms of creation and annihilation operators

(1) $$\phi \left(x,t\right)=\sum_{n}\left[{\hat{a}}_{n}^{\mathrm{in}}u_{n}\left(x,t\right)+{\hat{a}}_{n}^{†\;\mathrm{in}}u_{n}^{*}\left(x,t\right)\right],$$

where the mode functions $u_{n}\left(x,t\right)$ form a complete orthonormal set of solutions of the wave equation satisfying Dirichlet boundary conditions.

For t≤0, the cavity is static, and each field mode is given by

(2) $$u_{n}\left(x,t\right)=\frac{1}{\sqrt{2\omega_{n}}}\sqrt{\frac{2}{L_{x}}}\sin\left(\frac{n_{x}\pi }{L_{x}}x\right)\sqrt{\frac{2}{L_{y}}}\sin\left(\frac{n_{y}\pi }{L_{y}}y\right)\sqrt{\frac{2}{L_{z}}}\sin\left(\frac{n_{z}\pi }{L_{z}}z\right)e^{-i\omega_{k}t},$$

with

(3) $$\omega_{n}=\pi \sqrt{{\left(\frac{n_{x}}{L_{x}}\right)}^{2}\;+{\left(\frac{n_{y}}{L_{y}}\right)}^{2}\;+{\left(\frac{n_{z}}{L_{z}}\right)}^{2}}.$$

Here $n_{x},n_{y}$, and $n_{z}$ are positive integers and $n=(n_{x},n_{y},n_{z})$

In order to satisfy the boundary condition on the moving mirror, for 0<t<T, we expand the mode functions in Equation (1) using an “instantaneous basis”

(4) $$\begin{array}{ccc} u_{n}\left(x,t\right) & = & \sum_{k}Q_{k}^{\left(n\right)}\left(t\right)\sqrt{\frac{2}{L_{z}\left(t\right)}}\sin\left(\frac{k_{z}\pi }{L_{z}\left(t\right)}z\right)\sqrt{\frac{2}{L_{y}}}\sin\left(\frac{k_{y}\pi }{L_{y}}y\right)\sqrt{\frac{2}{L_{x}}}\sin\left(\frac{k_{x}\pi }{L_{x}}x\right) \end{array}$$

(5) $$\begin{array}{cc} = & \sum_{k}Q_{k}^{\left(n\right)}\left(t\right)\;\varphi_{k}\left(x,L_{z}\left(t\right)\right). \end{array}$$

The field degrees of freedom are now the functions $Q_{k}^{\left(n\right)}\left(t\right)$, that satisfy the initial conditions

(6) $$Q_{k}^{\left(n\right)}\left(0\right)=\frac{1}{\sqrt{2\omega_{n}}}\;\delta_{k,n}\;,\;\;{\dot{Q}}_{k}^{\left(n\right)}\left(0\right)=-i\sqrt{\frac{\omega_{n}}{2}}\;\delta_{k,n}.$$

The field modes $u_{n}\left(x,t\right)$ must satisfy the wave equation. Inserting the expansion (4) into the Klein Gordon equation, and taking into account that the $\varphi_{k}$’s form a complete and orthonormal set, one can obtain a set of coupled equations for $Q_{k}^{\left(n\right)}\left(t\right)$ [19]:

(7) $${\ddot{Q}}_{k}^{\left(n\right)}+\omega_{k}^{2}\left(t\right)\;Q_{k}^{\left(n\right)}=2\lambda \left(t\right)\sum_{j}g_{\mathrm{kj}}\;{\dot{Q}}_{j}^{\left(n\right)}+\dot{\lambda }\left(t\right)\sum_{j}g_{\mathrm{kj}}\;Q_{j}^{\left(n\right)}+\lambda^{2}\left(t\right)\sum_{j,l}g_{\mathrm{lk}}\;g_{\mathrm{lj}}\;Q_{j}^{\left(n\right)},$$

where

(8) $$\omega_{k}\left(t\right)=\pi \sqrt{{\left(\frac{k_{x}}{L_{x}}\right)}^{2}\;+{\left(\frac{k_{y}}{L_{y}}\right)}^{2}\;+{\left(\frac{k_{z}}{L_{z}\left(t\right)}\right)}^{2}}\;\;;\;\;\lambda \left(t\right)=\frac{{\dot{L}}_{z}\left(t\right)}{L_{z}\left(t\right)}.$$

The coefficients $g_{\mathrm{kj}}$ are defined by

(9) $$g_{\mathrm{kj}}=L_{z}\left(t\right)\int_{0}^{L_{z}\left(t\right)}dz\;\frac{\partial \varphi_{k}}{\partial L_{z}}\;\varphi_{j},$$

and read

(10) $$g_{\mathrm{kj}}=-g_{\mathrm{jk}}=\left\{\begin{array}{cc} {\left(-1\right)}^{k_{z}+j_{z}}\frac{2k_{z}j_{z}}{j_{z}^{2}-k_{z}^{2}}\;\delta_{k_{x}j_{x}}\;\delta_{k_{y}j_{y}}\; & \mathrm{if}\;k_{z}\neq j_{z}\\ 0 & \mathrm{if}\;k_{z}=j_{z}. \end{array}\right.$$

The annihilation and creation operators ${\hat{a}}_{k}^{\mathrm{in}}$ and ${\hat{a}}_{k}^{†\;\mathrm{in}}$ correspond to the particle notion in the ‘in’ region (t<0). When the wall stops for t>T, we can define a new set of operators, ${\hat{a}}_{k}^{\mathrm{out}}$ and ${\hat{a}}_{k}^{†\;\mathrm{out}}$, associated with the particle notion in the ‘out’ region. These two sets of operators are connected by a Bogoliubov transformation

(11) $${\hat{a}}_{k}^{\mathrm{out}}=\sum_{n}\left({\hat{a}}_{n}^{\mathrm{in}}\;\alpha_{\mathrm{nk}}+{\hat{a}}_{n}^{†\;\mathrm{in}}\;\beta_{\mathrm{nk}}^{⋆}\right).$$

The coefficients $\alpha_{\mathrm{nk}}$ and $\beta_{\mathrm{nk}}$ can be obtained from the solutions of the coupled equations (7). When the wall returns to its initial position, the solution reads

(12) $$Q_{k}^{\left(n\right)}\left(t\right)=A_{k}^{\left(n\right)}e^{-i\omega_{k}t}+B_{k}^{\left(n\right)}e^{i\omega_{k}t},$$

where $A_{k}^{\left(n\right)}$ and $B_{k}^{\left(n\right)}$ are constant coefficients to be determined by the continuity conditions at t=T. Inserting Equation (12) into Equations (1) and (4) we obtain an expansion of ϕ in terms of ${\hat{a}}_{k}^{\mathrm{in}}$ and ${\hat{a}}_{k}^{†\;\mathrm{in}}$ for t>T. Comparing this with the equivalent expansion in terms of ${\hat{a}}_{k}^{\mathrm{out}}$ and ${\hat{a}}_{k}^{†\;\mathrm{out}}$, it is easy to see that

(13) $$\alpha_{\mathrm{nk}}=\sqrt{2\omega_{k}}A_{k}^{\left(n\right)}\;\;,\;\;\beta_{\mathrm{nk}}=\sqrt{2\omega_{k}}\;B_{k}^{\left(n\right)}.$$

The number of particles created in the mode k is given by the mean value of the number operator ${\hat{a}}_{k}^{†\;\mathrm{out}}{\hat{a}}_{k}^{\mathrm{out}}$ with respect to the initial vacuum state (defined through ${\hat{a}}_{k}^{\mathrm{in}}|0_{\mathrm{in}}\rangle =0$). With the help of Equations (11) and (13), we get

(14) $$\langle 0_{\mathrm{in}}|N_{k}|0_{\mathrm{in}}\rangle =\langle 0_{\mathrm{in}}|{\hat{a}}_{k}^{†\;\mathrm{out}}{\hat{a}}_{k}^{\mathrm{out}}|0_{\mathrm{in}}\rangle =\sum_{n}2\omega_{k}|B_{k}^{\left(n\right)}{|}^{2}=\sum_{n}{\left|\beta_{\mathrm{nk}}\right|}^{2}.$$

All this formalism can be easily generalized for cylindrical cavities of arbitrary section in the $x_{⊥}=\left(x,y\right)$ plane. In this case, the instantaneous basis reads

(15) $$u_{n}\left(x,t\right)=\sum_{k_{z},k_{⊥}}Q_{k}^{\left(n\right)}\left(t\right)\sqrt{\frac{2}{L_{z}\left(t\right)}}\sin\left(\frac{k_{z}\pi }{L_{z}\left(t\right)}z\right)v_{k_{⊥}}\left(x_{⊥}\right)\;,$$

with $\nabla^{2}v_{k_{⊥}}=-k_{⊥}^{2}\;v_{k_{⊥}}$. For the scalar field considered in this paper, the transversal functions $v_{k_{⊥}}$ satisfy Dirichlet boundary conditions on the lateral surface of the cylinder. When describing the electromagnetic field in terms of Hertz potentials, the transverse electric potential is a scalar field that satisfies Neumann boundary conditions on the lateral surface, and Dirichlet boundary conditions on $z=0,\;z=L_{z}\left(t\right)$ [23]. In any case, it is interesting to remark that the coupling coefficients $g_{\mathrm{kj}}$ do not depend neither on the shape of the section of the cylinder nor on the boundary conditions on the the lateral surface.

## 3. Perturbative Evaluation of the Stochastic Particle Creation

In this section we solve Equation (7) perturbatively. We begin by writing these equations for a cavity of size $L_{z}\left(t\right)=L_{0}\left(1+\epsilon \xi \left(t\right)\right)$,

(16) $${\ddot{Q}}_{k}^{\left(n\right)}+\omega_{k}^{2}Q_{k}^{\left(n\right)}=2\epsilon \xi \left(t\right)\omega_{k_{z}}^{2}Q_{k}^{\left(n\right)}+2\epsilon \dot{\xi }\left(t\right)\sum_{m}g_{km}{\dot{Q}}_{m}^{\left(n\right)}+\epsilon \ddot{\xi }\left(t\right)\sum_{m}g_{km}Q_{m}^{\left(n\right)}\;,$$

where $\omega_{k_{z}}^{2}=k_{z}^{2}\pi^{2}/L_{0}^{2}$.

In order to use perturbation theory, we propose a solution of the form

(17) $$Q_{k}^{\left(n\right)}\left(t\right)=Q_{k}^{\left(n)(0\right)}\left(t\right)+\epsilon Q_{k}^{\left(n)(1\right)}\left(t\right),$$

and plug it into Equation (16). At the zeroth order in ϵ we have a simple harmonic oscillator, and the solution is

(18) $$Q_{k}^{\left(n)(0\right)}\left(t\right)=A_{k}^{\left(n\right)}e^{-i\omega_{k}t}+B_{k}^{\left(n\right)}e^{i\omega_{k}t},$$

where $A_{k}^{\left(n\right)}$ and $B_{k}^{\left(n\right)}$ are constants that will be fixed by the initial conditions. IIn the first order, we have

(19) $${\ddot{Q}}_{k}^{\left(n)(1\right)}+\omega_{k}^{2}Q_{k}^{\left(n)(1\right)}=2\xi \left(t\right)\omega_{k_{z}}^{2}Q_{k}^{\left(n)(0\right)}+2\dot{\xi }\left(t\right)\sum_{m}g_{km}{\dot{Q}}_{m}^{\left(n)(0\right)}+\ddot{\xi }\left(t\right)\sum_{m}g_{km}Q_{m}^{\left(n)(0\right)}.$$

Equation (19) corresponds to a driven harmonic oscillator, and can be solved using the corresponding Green’s function

(20) $$\begin{array}{cc} Q_{k}^{\left(n)(1\right)}\left(t\right) & =C_{k}^{\left(n\right)}e^{i\omega_{k}t}+D_{k}^{\left(n\right)}e^{-i\omega_{k}t}+\int_{0}^{\infty }dt^{\prime }\Theta \left(t-t^{\prime }\right)\frac{\sin[\omega_{k}\left(t-t^{\prime }\right)]}{\omega_{k}}\\ \; & \left[2\xi \left(t^{\prime }\right)\omega_{k_{z}}^{2}Q_{k}^{\left(n)(0\right)}\left(t^{\prime }\right)+2\dot{\xi }\left(t^{\prime }\right)\sum_{m}g_{km}{\dot{Q}}_{m}^{\left(n)(0\right)}\left(t^{\prime }\right)+\ddot{\xi }\left(t\right)\sum_{m}g_{km}Q_{m}^{\left(n)(0\right)}\left(t^{\prime }\right)\right]\;, \end{array}$$

where we added a homogeneous solution with arbitrary constants $C_{k}^{\left(n\right)}$ and $D_{k}^{\left(n\right)}$.

Replacing the result for $Q_{k}^{\left(n)(0\right)}$ and performing some integrations by parts, we find

(21) $$\begin{array}{cc} Q_{k}^{\left(n)(1\right)}\left(t\right) & =C_{k}^{\left(n\right)}e^{-i\omega_{k}t}+D_{k}^{\left(n\right)}e^{i\omega_{k}t}+2\omega_{k}\int_{0}^{T}dt^{\prime }\xi \left(t^{\prime }\right)\sin\left[\omega_{k}\left(t-t^{\prime }\right)\right]\\ \; & \sum_{m}\left(A_{k}^{\left(n\right)}e^{-i\omega_{k}t^{\prime }}+B_{k}^{\left(n\right)}e^{i\omega_{k}t^{\prime }}\right)\left(\delta_{mk}\frac{\omega_{k_{z}}^{2}}{\omega_{k}^{2}}+g_{km}\frac{\omega_{m}^{2}-\omega_{k}^{2}}{2\omega_{k}^{2}}\right), \end{array}$$

and, after imposing the initial conditions $B_{k}^{\left(n\right)}=C_{k}^{\left(n\right)}=D_{k}^{\left(n\right)}=0$ and $A_{k}^{\left(n\right)}=\frac{\delta_{n,k}}{\sqrt{2\omega_{k}}}$, we get

(22) $$\begin{array}{cc} Q_{k}^{\left(n\right)}\left(t\right) & =\frac{\delta_{kn}}{\sqrt{2\omega_{k}}}e^{-i\omega_{k}t}+\epsilon \frac{\sqrt{2\omega_{k}}}{\omega_{k}}v_{nk}\int_{0}^{T}dt^{\prime }\xi \left(t^{\prime }\right)\sin\left[\omega_{k}\left(t-t^{\prime }\right)\right]e^{-i\omega_{k}t^{\prime }}, \end{array}$$

where

(23) $$v_{nk}=\delta_{nk}\frac{\omega_{k_{z}}^{2}}{\omega_{k}}+g_{kn}\frac{\omega_{n}^{2}-\omega_{k}^{2}}{2\sqrt{\omega_{k}\omega_{n}}}.$$

The Bogoliubov coefficient can be obtained as follows

(24) $$\beta_{nk}=\left(i\omega_{k}Q_{k}^{\left(n\right)}\left(t\right)+{\dot{Q}}_{k}^{\left(n\right)}\left(t\right)\right)\frac{e^{-i\omega_{k}T}}{\sqrt{2\omega_{k}}}.$$

Therefore, replacing our perturbative solution for ${\dot{Q}}_{k}^{\left(n\right)}$, we get

(25) $$\beta_{nk}=-i\epsilon v_{nk}\int_{0}^{T}dt^{\prime }\;\xi \left(t^{\prime }\right)e^{-i\left(\omega_{n}+\omega_{k}\right)t^{\prime }}.$$

and

(26) $$|\beta_{nk}{|}^{2}=\epsilon^{2}v_{nk}^{2}\int_{0}^{T}\int_{0}^{T}dt^{\prime }dt^{\prime \prime }\xi \left(t^{\prime }\right)\xi \left(t^{\prime \prime }\right)e^{-i\left(\omega_{n}+\omega_{k}\right)\left(t^{\prime }-t^{\prime \prime }\right)}.$$

So far, we have not imposed any condition on $\xi (t^{\prime })$. Now, we study the case where we have a stochastic noise described by

(27) $$\langle \xi \left(t\right)\rangle =0\;,\;\;\langle \xi \left(t\right)\xi \left(t^{\prime }\right)\rangle =R\left(t-t^{\prime }\right)\;,$$

where R(t) is the noise correlation, and its Fourier transform is

(28) $$S\left(\nu \right)=\int_{0}^{\infty }R\left(u\right)e^{i\nu u}.$$

For this particular choice of ξ(t), we get

(29) $$\langle |\beta_{nk}{|}^{2}\rangle =2\epsilon^{2}Tv_{nk}^{2}ℜ\left[S\left(\omega_{n}+\omega_{k}\right)\right],$$

and therefore

(30) $$\langle N_{k}\rangle =2\epsilon^{2}T\sum_{n}v_{nk}^{2}ℜ\left[S\left(\omega_{n}+\omega_{k}\right)\right]\;.$$

This is the main result of this section. Note that the intermode coupling can be neglected only when the spectrum of the noise is sufficiently peaked around a single mode. To arrive at Equation (29), we assumed that ωT≫1, where ω represents the frequencies of the relevant modes in the cavity. However, the perturbative result will be valid as long as the higher-order corrections are smaller than the leading order, and this will be the case when $\epsilon^{2}\omega T≲1$.

It is interesting to compare the SPC with the deterministic counterpart. To do this, we come back to Equation (26) and assume that ξ(t) is a deterministic function. We see that $|\beta_{nk}{|}^{2}$ is proportional to $|\tilde{\xi }\left(\omega_{k}+\omega_{n}\right){|}^{2}$, where $\tilde{\xi }\left(\nu \right)$ is the Fourier transform of ξ(t). In particular, for an oscillatory motion ξ(t)=sin[Ωt] we obtain

(31) $$|\beta_{nk}{|}^{2}=\frac{1}{4}\epsilon^{2}v_{nk}^{2}T^{2}\delta_{\Omega ,\omega_{k}+\omega_{m}}\;.$$

We see that, for the stochastic motion, the total number of created particles grows as $\epsilon^{2}T$, for a deterministic, oscillatory motion, it grows as $\epsilon^{2}T^{2}$ [24]. These two time scales, ϵT and $\epsilon^{2}T$, will reappear in the nonperturbative calculations.

## 4. Non Perturbative Calculation: Single Mode

So far we have analyzed the system using a perturbative approach. However, this approach fails to describe the system at large timescales. One solution is to use the MSA, also called the “two times method“, which allows us to resume the essential contributions of a stochastic perturbation in order to obtain a uniformly valid solution [21,22].

In order to illustrate the method, we first obtain the solution to the equation of a harmonic oscillator Q(t) with multiplicative noise (stochastic time-dependent frequency) using MSA. Then we apply a similar approach to compute ${\left|Q\right|}^{2}$, that allows us to obtain the squared modulus of the Bogoliubov coefficients.

Before doing this, we remind the reader that, for a harmonic oscillator with a deterministic time-dependent frequency,

(32) $$\ddot{Q}+\omega^{2}\left[1+\epsilon \sin\left[2\omega t\right]\right]Q=0,$$

the naive perturbative expansion in powers of ϵ contains secular terms that are $O({\left(\epsilon \omega t\right)}^{n})$, where *n* is the order of the approximation. The perturbative solution is therefore valid at short times ϵωt≪1. To remediate this, one introduces a second timescale $\bar{\tau }=\epsilon t$ and expands the solution as

(33) $$Q\left(t\right)=Y^{\left(0\right)}\left(t,\bar{\tau }\right)+\epsilon Y^{\left(1\right)}\left(t,\bar{\tau }\right)+O\left(\epsilon^{2}\right).$$

The functions $Y^{\left(0\right)}$ and $Y^{\left(1\right)}$ satisfy

(34) $$\begin{array}{c} \partial_{t}^{2}Y^{\left(0\right)}+\omega^{2}Y^{\left(0\right)}=0,\\ \partial_{t}^{2}Y^{\left(1\right)}+\omega^{2}Y^{\left(1\right)}=-\omega^{2}\sin\left[2\omega t\right]Y^{\left(0\right)}-2\partial_{t\bar{\tau }}^{2}Y^{\left(0\right)}\;. \end{array}$$

The leading order solution reads

(35) $$Y^{\left(0\right)}\left(t,\bar{\tau }\right)=A\left(\bar{\tau }\right)e^{-i\omega t}+B\left(\bar{\tau }\right)e^{i\omega t},$$

and the functions $A(\bar{\tau })$ and $B(\bar{\tau })$ are chosen such that there are no secular terms in $Y^{\left(1\right)}\left(t,\bar{\tau }\right)$; that is, no terms proportional to $e^{\pm i\omega t}$ in the r.h.s. of Equation (34). A simple calculation gives

(36) $$\frac{dA}{d\bar{\tau }}=\frac{\omega }{4}B\;,\;\;\frac{dB}{d\bar{\tau }}=\frac{\omega }{4}A\;.$$

Therefore, with the initial conditions $A\left(0\right)=1/\sqrt{2\omega }\;,B\left(0\right)=0$ we obtain

(37) $$A\left(\bar{\tau }\right)=\frac{1}{\sqrt{2\omega }}\cosh\left[\frac{\omega }{4}\bar{\tau }\right]\;,\;\;B\left(\bar{\tau }\right)=\frac{1}{\sqrt{2\omega }}\sinh\left[\frac{\omega }{4}\bar{\tau }\right]\;,$$

and

(38) $${\left|\beta \right|}^{2}=\sinh^{2}\left[\frac{\omega }{4}\bar{\tau }\right]={\left|\alpha \right|}^{2}-1\;.$$

This shows the amplification of the oscillations due to parametric resonance. We turn now to the stochastic case.

### 4.1. MSA for a Stochastic Harmonic Oscillator

We start our discussion of the stochastic MSA by studying a simple example, a random harmonic oscillator, given by

(39) $$\ddot{Q}+\omega^{2}\left[1+\epsilon \xi \left(t\right)\right]Q=0,$$

with initial conditions

(40) $$Q\left(0\right)=\frac{1}{\sqrt{2\omega }},\;\;\;\;\dot{Q}\left(0\right)=-i\sqrt{\frac{\omega }{2}},$$

where the frequency has a stochastic component given by ξ(t). A similar problem can be found in [22]. As before, the noise is characterized by its mean value and its correlation function $R(t-t^{\prime })$, see Equation (27).

Following the MSA method [19,21], we introduce the new timescale $\tau =\epsilon^{2}t$, and expand our solution in powers of ϵ as follows

(41) $$Q\left(t\right)=Y^{\left(0\right)}\left(t,\tau \right)+\epsilon Y^{\left(1\right)}\left(t,\tau \right)+\epsilon^{2}Y^{\left(2\right)}\left(t,\tau \right)+O\left(\epsilon^{3}\right).$$

Introducing this expansion in Equation (39), we get the set of equations

(42) $$\begin{array}{ccc} & & \partial_{t}^{2}Y^{\left(0\right)}+\omega^{2}Y^{\left(0\right)}=0,\\ & & \partial_{t}^{2}Y^{\left(1\right)}+\omega^{2}Y^{\left(1\right)}+\omega^{2}\xi \left(t\right)Y^{\left(0\right)}=0,\\ & & \partial_{t}^{2}Y^{\left(2\right)}+\omega^{2}Y^{\left(2\right)}+\omega^{2}\xi \left(t\right)Y^{\left(1\right)}+2\partial_{\tau t}^{2}Y^{\left(0\right)}=0. \end{array}$$

First, we notice that $Y^{\left(0\right)}$ satisfies the equation of a simple harmonic oscillator of frequency ω, so the solution to the first equation is

(43) $$Y^{\left(0\right)}\left(t,\tau \right)=A\left(\tau \right)e^{-i\omega t}+B\left(\tau \right)e^{i\omega t},$$

where A(τ) and B(τ) are the slowly varying functions we want to determine. These functions will be fixed by imposing that the stochastic mean values $\langle Y^{\left(1\right)}\rangle$ and $\langle Y^{\left(2\right)}\rangle$ do not have secular terms.

The second equation in (42) is that of a harmonic oscillator with a source $-\omega^{2}\xi \left(t\right)Y^{\left(0\right)}$. To solve this equation, we use the harmonic oscillator propagator to compute the contribution of the source, and the solution (43), obtaining

(44) $$Y^{\left(1\right)}\left(t,\tau \right)=C\left(\tau \right)e^{-i\omega t}+D\left(\tau \right)e^{i\omega t}-\omega \int_{0}^{t}dt^{\prime }\sin\left[\omega \left(t-t^{\prime }\right)\right]\xi \left(t^{\prime }\right)\left[A\left(\tau \right)e^{-i\omega t^{\prime }}+B\left(\tau \right)e^{i\omega t^{\prime }}\right],$$

where C(τ) and D(τ) are arbitary functions. Note that the τ under the integral sign has not been integrated since we are solving partial differential equations where τ and *t* are independent variables. Furthermore, averaging over the noise, we find that

(45) $$\langle Y^{\left(1\right)}\left(t,\tau \right)\rangle =C\left(\tau \right)e^{-i\omega t}+D\left(\tau \right)e^{i\omega t}.$$

Being linear in the noise, the source has vanishing mean value and $\langle Y^{\left(1\right)}\rangle$ has no secular terms. Therefore, there are no conditions imposed on A(τ) or B(τ) yet. As a consequence, it is necessary to consider the terms of order $O(\epsilon^{2})$. This is also the reason why the new timescale for a stochastic frequency is $\tau =\epsilon^{2}t$ and not $\bar{\tau }=\epsilon t$, as in the deterministic case.

Solving the last equation in (42) by the same method we get

(46) $$\begin{array}{cc} Y^{\left(2\right)}\left(t,\tau \right)= & E\left(\tau \right)e^{-i\omega t}+F\left(\tau \right)e^{i\omega t}+2i\int_{0}^{t}dt^{\prime }\sin\left[\omega \left(t-t^{\prime }\right)\right]\left[A^{\prime }\left(\tau \right)e^{-i\omega t^{\prime }}-B^{\prime }\left(\tau \right)e^{i\omega t^{\prime }}\right]\\ & -\omega \int_{0}^{t}dt^{\prime }\sin\left[\omega \left(t-t^{\prime }\right)\right]\xi \left(t^{\prime }\right)\{C\left(\tau \right)e^{-i\omega t^{\prime }}+D\left(\tau \right)e^{i\omega t^{\prime }}\\ & -\omega \int_{0}^{t^{\prime }}dt^{\prime \prime }\sin\left[\omega \left(t^{\prime }-t^{\prime \prime }\right)\right]\xi \left(t^{\prime \prime }\right)\left[A\left(\tau \right)e^{-i\omega t^{\prime \prime }}+B\left(\tau \right)e^{i\omega t^{\prime \prime }}\right]\}, \end{array}$$

where a prime denotes derivative with respect to τ, and E(τ) and F(τ) are arbitrary functions. Now, if we take the mean value of $Y^{\left(2\right)}$ we obtain

(47) $$\begin{array}{cc} \langle Y^{\left(2\right)}\left(t,\tau \right)\rangle = & E\left(\tau \right)e^{-i\omega t}+F\left(\tau \right)e^{i\omega t}+\int_{0}^{t}dt^{\prime }\sin\left[\omega \left(t-t^{\prime }\right)\right]\{2i\left[A^{\prime }\left(\tau \right)e^{-i\omega t^{\prime }}-B^{\prime }\left(\tau \right)e^{i\omega t^{\prime }}\right]\\ \; & +\omega^{2}\int_{0}^{t^{\prime }}dt^{\prime \prime }\sin\left[\omega \left(t^{\prime }-t^{\prime \prime }\right)\right]R\left(t^{\prime }-t^{\prime \prime }\right)\left[A\left(\tau \right)e^{-i\omega t^{\prime \prime }}+B\left(\tau \right)e^{i\omega t^{\prime \prime }}\right]\}, \end{array}$$

that can be rewritten as

(48) $$\begin{array}{cc} \langle Y^{\left(2\right)}\left(t,\tau \right)\rangle = & E\left(\tau \right)e^{-i\omega t}+F\left(\tau \right)e^{i\omega t}+\int_{0}^{t}dt^{\prime }\sin\left[\omega \left(t-t^{\prime }\right)\right]\{2i\left[A^{\prime }\left(\tau \right)e^{-i\omega t^{\prime }}-B^{\prime }\left(\tau \right)e^{i\omega t^{\prime }}\right]\\ \; & +\omega^{2}\int_{0}^{t^{\prime }}du\sin\left[\omega u\right]R\left(u\right)\left[A\left(\tau \right)e^{-i\omega (t^{\prime }-u)}+B\left(\tau \right)e^{i\omega (t^{\prime }-u)}\right]\}\;. \end{array}$$

We notice that there are secular terms, i.e., terms proportional to $e^{\pm i\omega t^{\prime }}$ inside the $t^{\prime }$-integral. So we choose A(τ) and B(τ) such that these secular terms vanish. Therefore, we set

(49) $$\begin{array}{cc} 0= & e^{-i\omega t^{\prime }}\left[A^{\prime }\left(\tau \right)-A\left(\tau \right)\frac{\omega^{2}}{4}\int_{0}^{t^{\prime }}duR\left(u\right)-B\left(\tau \right)e^{2i\omega t^{\prime }}\int_{0}^{t^{\prime }}duR\left(u\right)e^{-2i\omega u}\right]\\ \; & +e^{i\omega t^{\prime }}\left[B^{\prime }\left(\tau \right)+B\left(\tau \right)\frac{\omega^{2}}{4}\int_{0}^{t^{\prime }}duR\left(u\right)+A\left(\tau \right)e^{-2i\omega t^{\prime }}\int_{0}^{t^{\prime }}duR\left(u\right)e^{2i\omega u}\right]. \end{array}$$

In terms of the Fourier transform of the correlation Equation (28) we get, in the large $t^{\prime }$ limit

(50) $$\begin{array}{ccc} A^{\prime }\left(\tau \right) & = & \frac{\omega^{2}}{4}\left[S(2\omega )-S(0)\right]A\left(\tau \right)\\ B^{\prime }\left(\tau \right) & = & \frac{\omega^{2}}{4}\left[S{\left(2\omega \right)}^{*}-S\left(0\right)\right]B\left(\tau \right). \end{array}$$

Solving these equations with the initial conditions Equation (40) we obtain

(51) $$Q\left(t\right)=\frac{1}{\sqrt{2\omega }}e^{-i\omega t}e^{\frac{\omega^{2}}{4}\left[S(2\omega )-S(0)\right]\epsilon^{2}t}+O\left(\epsilon \right)\;.$$

We see that the solution has an exponential growth as long as ℜ[S(2ω)−S(0)]>0. This is a stochastic counterpart of the deterministic parametric resonance. The noise also produces a shift in the frequency of oscillation: $\omega \to \omega -\epsilon^{2}\omega^{2}ℑ\left[S\left(2\omega \right)\right]/4$.

Notice that we only used the expansion in ϵ to find the conditions on the slowly varying functions A(τ) and B(τ) that ensure vanishing secular terms in the lowest order of the expansion. This is the main idea of the MSA. Moreover, the solution we have found, $Q\left(t\right)≃Y^{\left(0\right)}$, is valid to the lowest order in ϵ. However, as we know that $\langle Y^{\left(1\right)}\rangle =0$, the mean value of Q(t) satisfies

(52) $$\langle Q\left(t\right)\rangle =\frac{1}{\sqrt{2\omega }}e^{-i\omega t}e^{\frac{\omega^{2}}{4}\left[S(2\omega )-S(0)\right]\epsilon^{2}t}+O\left(\epsilon^{2}\right)\;.$$

Had we solved the stochastic differential equation for Q(t) with the initial conditions

(53) $$Q\left(0\right)=1,\;\;\;\;\dot{Q}\left(0\right)=0\;,$$

we would obtain

(54) $$\langle Q\left(t\right)\rangle =e^{\frac{\omega^{2}}{4}\left[ℜ[S(2\omega )]-S(0)\right]\epsilon^{2}t}\cos\left[\left(\omega -\frac{\omega^{2}\epsilon^{2}}{4}ℑ\left[S\left(2\omega \right)\right]\right)t\right]+O\left(\epsilon^{2}\right)\;,$$

which coincides with the result obtained in Ref. [22] using a different approach.

### 4.2. Bogoliubov Coefficients Using Msa

If the random motion stops at t=T, for t>T we will have

(55) $$Q\left(t\right)=\frac{\alpha }{\sqrt{2\omega }}e^{-i\omega t}+\frac{\beta }{\sqrt{2\omega }}e^{i\omega t}\;,$$

where α and β are the Bogoliubov coefficients. One could read the stochastic mean values of α and β from Equation (52). However, the number of created particles is given by $\langle |\beta {|}^{2}\rangle$ and, as Q(t) is a stochastic variable, one cannot obtain $\langle |\beta {|}^{2}\rangle$ from 〈Q(t)〉. Our strategy to compute $\langle |\beta {|}^{2}\rangle$ will be to implement the MSA imposing that $\langle |Q\left(t\right){|}^{2}\rangle$ does not include secular terms. Taking into account that, for t>T,

(56) $${\left|Q\left(t\right)\right|}^{2}=\frac{1}{2\omega }\left({\left|\alpha \right|}^{2}+{\left|\beta \right|}^{2}+2ℜ\left[\alpha \beta^{*}e^{-2i\omega t}\right]\right)\;,$$

and that ${\left|\alpha \right|}^{2}-{\left|\beta \right|}^{2}=1$, we will be able to compute $\langle |\beta {|}^{2}\rangle$ from the non-oscillating part of $\langle |Q\left(t\right){|}^{2}\rangle$.

From Equation (41), we have

(57) $${\langle |Q\left(t\right)|}^{2}\rangle =|Y^{\left(0\right)}{|}^{2}+\epsilon^{2}\langle 2ℜ\left[Y^{(0)*}Y^{\left(2\right)}\right]+|Y^{\left(1\right)}{|}^{2}\rangle \;.$$

In the previous section, we determined A(τ) and B(τ) from the absence of secular terms in $\langle Y^{\left(2\right)}\rangle$. Here, these functions will be such that no secular terms appear in

(58) $$2ℜ\left[Y^{(0)*}\langle Y^{\left(2\right)}\rangle \right]+\langle |Y^{\left(1\right)}{|}^{2}\rangle .$$

We have already computed $\langle Y^{\left(2\right)}\rangle$ in Equation (48). The mean value $\langle |Y^{\left(1\right)}{|}^{2}\rangle$ can be obtained from Equation (44) and reads

(59) $$\begin{array}{ccc} \langle |Y^{\left(1\right)}{|}^{2}\rangle & = & {\left|C\right|}^{2}+{\left|D\right|}^{2}+2ℜ\left[C^{*}De^{2i\omega t}\right]+\omega^{2}\int_{0}^{t}dt^{\prime }\int_{0}^{t}\sin\left[\omega \left(t-t^{\prime }\right)\right]dt^{\prime \prime }\sin\left[\omega \left(t-t^{\prime \prime }\right)\right]\\ & & \times R\left(t^{\prime }-t^{\prime \prime }\right)\left(Ae^{-i\omega t^{\prime }}+Be^{i\omega t^{\prime }}\right)\left(A^{*}e^{i\omega t^{\prime \prime }}+B^{*}e^{-i\omega t^{\prime \prime }}\right)\;. \end{array}$$

In order to isolate the secular terms, it is useful to note that, for a function $f(t,t^{\prime })$ such that $f\left(t^{\prime },t^{\prime \prime }\right)=f^{*}\left(t^{\prime \prime },t^{\prime }\right)$, we have

(60) $$\begin{array}{ccc} & & \int_{0}^{t}dt^{\prime }\int_{0}^{t}dt^{\prime \prime }\;f\left(t^{\prime },t^{\prime \prime }\right)=2ℜ\left[\int_{0}^{t}dt^{\prime }\int_{0}^{t^{\prime }}dt^{\prime \prime }\;f\left(t^{\prime },t^{\prime \prime }\right)\right]\\ & & 2ℜ\left[\int_{0}^{t}dt^{\prime }\int_{0}^{t^{\prime }}du\;f\left(t^{\prime },t^{\prime }-u\right)\right]\;. \end{array}$$

Using this property in Equation (59), inserting the result into Equation (58) and using Equation (48), we find, after a straightforward calculation, the differential equations that must satisfy A(τ) and B(τ) to cancel the secular terms in $\langle |Q\left(t\right){|}^{2}\rangle$. They are given by

(61) $$\begin{array}{ccc} & & {\left(|A\left(\tau \right)\right|}^{2}+{\left|B\left(\tau \right)\right|}^{2}{)}^{\prime }=\omega^{2}{ℜ\left[S\left(2\omega \right)\right](|A\left(\tau \right)|}^{2}+{\left|B\left(\tau \right)\right|}^{2}),\\ & & {\left(A^{*}\left(\tau \right)B\left(\tau \right)\right)}^{\prime }=\frac{\omega^{2}}{2}\left(S^{*}\left(2\omega \right)-2S\left(0\right)\right)A^{*}\left(\tau \right)B\left(\tau \right)\;. \end{array}$$

At this point it is worth remarking that the functions A(τ) and B(τ) that cancel the secular terms in $\langle |Q\left(t\right){|}^{2}\rangle$ are different from those that cancel the secular terms in 〈Q(t)〉. This is of course due to the fact that we are dealing with a stochastic differential equation, and therefore ${\left|\langle Q\left(t\right)\rangle \right|}^{2}\neq {\langle |Q\left(t\right)|}^{2}\rangle$.

Note that the two differentials in Equation (61) are enough to determine $\langle |Q\left(t\right){|}^{2}\rangle$, given by

(62) $${\langle |Q\left(t\right)|}^{2}\rangle =|Y^{\left(0\right)}{|}^{2}+O\left(\epsilon^{2}\right)=|A\left(\tau \right){|}^{2}+{\left|B\left(\tau \right)\right|}^{2}+2ℜ\left[A^{*}\left(\tau \right)B\left(\tau \right)e^{2i\omega t}\right]+O\left(\epsilon^{2}\right)\;.$$

Using the adequate initial conditions $A\left(0\right)=1/\sqrt{2\omega }$, B(0)=0, we find for the Bogoliubov coefficient,

(63) $${\langle |\beta |}^{2}\rangle =\frac{1}{2}\left(e^{\omega^{2}ℜ\left[S\left(2\omega \right)\right]\epsilon^{2}t}-1\right)\;.$$

This is the main result of this section: the stochastic mean value of the modulus squared of the Bogoliubov coefficient grows exponentially with a rate $\epsilon^{2}\omega^{2}ℜ\left[S\left(2\omega \right)\right]$.

As a test of our approach, we make contact with previous results in the literature. The procedure described above can be applied to compute $\langle Q^{2}\left(t\right)\rangle$ instead of $\langle |Q\left(t\right){|}^{2}\rangle$. Doing this, with the initial conditions Q(0)=1 and $\dot{Q}\left(0\right)=0$, we obtain

(64) $$\begin{array}{ccc} \langle Q^{2}\left(t\right)\rangle & = & \frac{1}{2}e^{\frac{\omega^{2}}{2}\left[ℜ[S(2\omega )]-2S(0)\right]\epsilon^{2}t}\cos\left[\left(2\omega -\frac{\omega^{2}\epsilon^{2}}{2}ℑ\left[S\left(2\omega \right)\right]\right)t\right]\\ & + & \frac{1}{2}e^{\omega^{2}ℜ\left[S\left(2\omega \right)\right]\epsilon^{2}t}+O\left(\epsilon^{2}\right)\;, \end{array}$$

that coincides with the result obtained in Ref. [22]. Equation (64) can be obtained from Equation (62) with the initial conditions A(0)=B(0)=1/2.

We can apply the analysis of this section to the case of DCE in a cubic cavity. In the single-mode approximation, we set $g_{\mathrm{kj}}=0$ in Equation (16). The resulting equation is of the form of Equation (39). Therefore, the number of created particles due to the stochastic motion of one of its walls reads

(65) $$\langle |\beta_{k}{|}^{2}\rangle =\frac{1}{2}\left(e^{\frac{4\omega_{k_{z}}^{4}}{\omega_{k}^{2}}ℜ\left[S\left(2\omega_{k}\right)\right]\epsilon^{2}t}-1\right)\;.$$

We see the different time scales in the rate of particle creation in the stochastic and deterministic situations: $\tau =\epsilon^{2}t$ in the former and $\bar{\tau }=\epsilon t$ in the latter. The MSA results are compatible with the perturbative results discussed at the end of Section 3: at short times, Equation (65) is proportional to $\epsilon^{2}t$, while from Equation (38), we see that the square of the modulus of the β coefficient in the deterministic case is proportional to $\epsilon^{2}t^{2}$.

Although the single-mode approximation is a typical approximation in quantum optics, the perturbative calculations of Section 3 suggest that it is not justified for a noisy excitation of the system. In the next section we relax the single mode assumption and consider the case in which there is intermode coupling.

## 5. Nonperturbative Calculation: Coupled Modes

In order to assess the relevance of intermode couplings we analyze the solutions of Equation (16) using MSA. As before, we first compute the stochastic mean value $\langle Q_{k}^{\left(n\right)}\rangle$ and then the Bogoliubov coefficients. In order to simplify the notation, we omit the supraindex (n) in the intermediate calculations.

Using the expansion

(66) $$Q_{k}\left(t\right)=Y_{k}^{\left(0\right)}\left(t,\tau \right)+\epsilon Y_{k}^{\left(1\right)}\left(t,\tau \right)+\epsilon^{2}Y_{k}^{\left(2\right)}\left(t,\tau \right)+O\left(\epsilon^{3}\right)\;,$$

we obtain, from Equation (16),

(67) $$\begin{array}{ccc} & & \partial_{t}^{2}Y_{k}^{\left(0\right)}+\omega_{k}^{2}Y_{k}^{\left(0\right)}=0\;, \end{array}$$

(68) $$\begin{array}{ccc} & & \partial_{t}^{2}Y_{k}^{\left(1\right)}+\omega_{k}^{2}Y_{k}^{\left(1\right)}=2\omega_{k_{z}}^{2}\xi \left(t\right)Y_{k}^{\left(0\right)}+2\dot{\xi }\sum_{m}g_{km}\partial_{t}Y_{m}^{\left(0\right)}+\ddot{\xi }\sum_{m}g_{km}Y_{m}^{\left(0\right)}\;, \end{array}$$

(69) $$\begin{array}{ccc} & & \partial_{t}^{2}Y_{k}^{\left(2\right)}+\omega_{k}^{2}Y_{k}^{\left(2\right)}=-2\partial_{\tau t}^{2}Y_{k}^{\left(0\right)}+2\dot{\xi }\sum_{m}g_{km}\partial_{t}Y_{m}^{\left(1\right)}+\ddot{\xi }\sum_{m}g_{km}Y_{m}^{\left(1\right)}+\omega_{k_{z}}^{2}\xi Y_{k}^{\left(1\right)}\;. \end{array}$$

The zeroth-order solution is

(70) $$Y_{k}^{\left(0\right)}\left(t,\tau \right)=A_{k}\left(\tau \right)e^{-i\omega_{k}t}+B_{k}\left(\tau \right)e^{i\omega_{k}t},$$

and the first- and second-order solutions (l=1,2) are

(71) $$Y_{k}^{\left(l\right)}\left(t,\tau \right)=\int_{0}^{t}dt^{\prime }\;\frac{\sin[\omega_{k}\left(t-t^{\prime }\right)]}{\omega_{k}}J_{k}^{\left(l\right)}\left(t^{\prime },\tau \right)\;,$$

where we have introduced the notation $J_{k}^{\left(1\right)}\left(t,\tau \right)$ and $J_{k}^{\left(2\right)}\left(t,\tau \right)$ for the r.h.s. of Equations (68) and (69), respectively.

### 5.1. Msa for Coupled Oscillators

As before, the slowly varying functions $A_{k}\left(\tau \right)$ and $B_{k}\left(\tau \right)$ are fixed by imposing that $\langle Q_{k}\left(t\right)\rangle$ does not have secular terms, that is, $\langle J_{k}^{\left(l\right)}\left(t^{\prime },\tau \right)\rangle$ should not have terms proportional to $e^{\pm i\omega_{k}t}$ (see Equation (71)). Being linear in the noise, $\langle J_{k}^{\left(1\right)}\left(t^{\prime },\tau \right)\rangle$ vanishes; only $\langle J_{k}^{\left(2\right)}\left(t^{\prime },\tau \right)\rangle$ is relevant.

We illustrate the calculation with the first two terms in $\langle J_{k}^{\left(2\right)}\left(t^{\prime },\tau \right)\rangle$, that are $\left(a\right)\equiv -2\partial_{\tau t}^{2}Y_{k}^{\left(0\right)}\left(t^{\prime },\tau \right)$ and $\left(b\right)\equiv 2\langle \dot{\xi }\sum_{m}g_{km}\partial_{t}Y_{m}^{\left(1\right)}\left(t^{\prime },\tau \right)\rangle$. The first term does not have a stochastic contribution and reads

(72) $$\left(a\right)=2i\omega_{k}\left[A_{k}^{\prime }e^{-i\omega_{k}t}-B_{k}^{\prime }e^{i\omega_{k}t}\right]\;.$$

The evaluation of the second term is more complex. We have

(73) $$\begin{array}{ccc} \left(b\right) & = & 2\sum_{m}g_{km}\int_{0}^{t^{\prime }}\cos[\omega_{k}\left(t^{\prime }-t^{\prime \prime }\right]\{2\omega_{k_{z}}^{2}\langle \dot{\xi }\left(t^{\prime }\right)\xi \left(t^{\prime \prime }\right)\rangle Y_{m}^{\left(0\right)}\left(t^{\prime \prime },\tau \right)\\ & & +2\langle \dot{\xi }\left(t^{\prime }\right)\dot{\xi }\left(t^{\prime \prime }\right)\rangle \sum_{n}g_{kn}\partial_{t}Y_{n}^{\left(0\right)}\left(t^{\prime \prime },\tau \right)+\langle \dot{\xi }\left(t^{\prime }\right)\ddot{\xi }\left(t^{\prime \prime }\right)\rangle \sum_{n}g_{kn}Y_{n}^{\left(0\right)}\left(t^{\prime \prime },\tau \right)\}. \end{array}$$

As the noise correlation functions depend on $t^{\prime }-t^{\prime \prime }$, the $t^{\prime }$-integral in Equation (73) is a sum of terms of the form

(74) $$I_{nk}=\int_{0}^{t^{\prime }}du\;\cos\left[\omega_{k}u\right]\left[G\left(u\right)A_{n}e^{-i\omega_{n}\left(t^{\prime }-u\right)}+G^{*}\left(u\right)B_{n}e^{i\omega_{n}\left(t^{\prime }-u\right)}\right]\;,$$

where the function G(u) has the information of the correlation functions. Therefore

(75) $$2I_{nk}=A_{n}\left[\tilde{G}\left(\omega_{n}+\omega_{k}\right)+\tilde{G}\left(\omega_{n}-\omega_{k}\right)\right]e^{-i\omega_{n}t^{\prime }}+B_{n}\left[\tilde{G}\left(\omega_{-n}+\omega_{k}\right)+\tilde{G}\left(-\omega_{n}-\omega_{k}\right)\right]e^{i\omega_{n}t^{\prime }}\;,$$

where we introduced the notation

(76) $$\tilde{G}\left(\nu \right)=\int_{0}^{\infty }duG\left(u\right)e^{iu\nu }\;.$$

From these expressions is easy to recognize the secular terms, that will depend on the Fourier transform of the correlation functions evaluated at $\pm \omega_{k}\pm \omega_{n}$.

Following this procedure one can obtain the differential equations that determine the functions $A_{k}\left(\tau \right)$ and $B_{k}\left(\tau \right)$. The result is

(77) $$\begin{array}{ccc} & & A_{k}^{\prime }\left(\tau \right)+\lambda_{k}A_{k}\left(\tau \right)=0\\ & & B_{k}^{\prime }\left(\tau \right)+\lambda_{k}^{*}B_{k}\left(\tau \right)=0\;, \end{array}$$

with

(78) $$\lambda_{k}=\frac{\omega_{k_{z}}^{4}}{\omega_{k}^{2}}\left(S\left(0\right)-S\left(2\omega_{k}\right)\right)-\sum_{m}\frac{g_{km}^{2}}{4\omega_{k}\omega_{m}}{\left(\omega_{k}^{2}-\omega_{m}^{2}\right)}^{2}\left(S\left(\omega_{k}+\omega_{n}\right)-S\left(\omega_{k}-\omega_{n}\right)\right)\;.$$

Note that while the rate of change $\lambda_{k}$ depend on the intermode coupling constants $g_{km}$, the differential equations for the modes are uncoupled. This is somewhat unexpected. However, a closer look at the equations reveals that this should be the case for a nondegenerate spectrum because of the MSA condition, i.e., the absence of secular terms in $\langle J_{k}^{\left(2\right)}\left(t^{\prime },\tau \right)\rangle$, is linear in the zeroth-order solution $Y_{m}^{\left(0\right)}$. Therefore, the MSA condition selects the terms with m=k if the spectrum is nondegenerate.

Restoring the supraindex (n), and taking into account the initial conditions, the MSA solution for $\langle Q_{k}^{\left(n\right)}\rangle$ reads

(79) $$\langle Q_{k}^{\left(n\right)}\rangle =\frac{\delta_{k,n}}{\sqrt{2\omega_{k}}}e^{-i\omega_{k}t-\epsilon^{2}\lambda_{k}t}+O\left(\epsilon^{2}\right)\;.$$

### 5.2. The Bogoliubov Coefficients from Msa

The calculation of the mean value of the modulus squared of the Bogoliubov coefficients is more complicated than in the case of uncoupled oscillators. The reason is the following. The generalization of Equation (56) is, in this case

(80) $$|Q_{k}^{\left(n\right)}{|}^{2}=\frac{1}{2\omega_{k}}\left(|\alpha_{n}{k|}^{2}+{\left|\beta_{n}k\right|}^{2}+2ℜ\left[\alpha_{n}k\beta_{n}k^{*}e^{-2i\omega_{k}t}\right]\right)\;.$$

Taking this into account, in the MSA approximation,

(81) $$\langle |Q_{k}^{\left(n\right)}{|}^{2}\rangle =\left(|A_{k}^{\left(n\right)}{|}^{2}+{\left|B_{k}^{\left(n\right)}\right|}^{2}+2ℜ\left[A_{k}^{\left(n\right)}B_{k}^{(n)*}e^{-2i\omega_{k}t}\right]\right)+O\left(\epsilon^{2}\right)\;,$$

one can read the combination $\langle |\alpha_{\mathrm{nk}}{|}^{2}+|\beta_{\mathrm{nk}}{|}^{2}\rangle$ from the MSA solution. The additional complication comes from the fact that, for a single mode, one has the condition ${\left|\alpha \right|}^{2}-{\left|\beta \right|}^{2}=1$; for coupled oscillators, the Bogoliubov coefficients satisfy

(82) $$\sum_{k}\left(|\alpha_{n}{k|}^{2}-{\left|\beta_{n}k\right|}^{2}\right)=1\;.$$

As a consequence, it is not possible to obtain both $|\alpha_{\mathrm{nk}}{|}^{2}$ and $|\beta_{\mathrm{nk}}{|}^{2}$ from the MSA solution for $\langle |Q_{k}^{\left(n\right)}{|}^{2}\rangle$ alone, as in the single-mode case.

To proceed, one can consider the additional quantity

(83) $$Q_{k}^{\left(n\right)}{\dot{Q}}_{k}^{(n)*}=\frac{i}{2}\left(|\alpha_{n}{k|}^{2}-{\left|\beta_{n}k\right|}^{2}-2iℑ\left[\alpha_{n}k\beta_{n}k^{*}e^{-2i\omega_{k}t}\right]\right)\;,$$

and therefore compute $\langle |\alpha_{\mathrm{nk}}{|}^{2}-|\beta_{\mathrm{nk}}{|}^{2}\rangle$ from $\langle Q_{k}^{\left(n\right)}{\dot{Q}}_{k}^{(n)*}\rangle$. The procedure to determine $\langle |\alpha_{\mathrm{nk}}{|}^{2}\rangle$ and $\langle |\beta_{\mathrm{nk}}{|}^{2}\rangle$ is then to calculate both $\langle |Q_{k}^{\left(n\right)}{|}^{2}\rangle$ and $\langle Q_{k}^{\left(n\right)}{\dot{Q}}_{k}^{(n)*}\rangle$ using the MSA, imposing, in each case, the absence of secular terms.

Here, we sketch the computation of the *total* number of created particles, that can be obtained from $\langle |Q_{k}^{\left(n\right)}{|}^{2}\rangle$. Defining

(84) $$T_{k}^{\left(n\right)}\left(\tau \right)=|A_{k}^{\left(n\right)}{\left(\tau \right)|}^{2}+{\left|B_{k}^{\left(n\right)}\left(\tau \right)\right|}^{2}\;,$$

after a long calculation one can show that, to avoid the secular terms in $\langle |Q_{k}^{\left(n\right)}{|}^{2}\rangle$, $T_{k}^{\left(n\right)}$ must satisfy the differential equation

(85) $$T_{k}^{{\left(n\right)}^{\prime }}\left(\tau \right)+\gamma_{k}T_{k}^{\left(n\right)}\left(\tau \right)+\sum_{m}\rho_{mk}T_{m}^{\left(n\right)}\left(\tau \right)=0\;,$$

where

(86) $$\gamma_{k}=-4\frac{\omega_{k_{z}}^{4}}{\omega_{k}^{2}}ℜ\left[S\left(2\omega_{k}\right)\right]-\sum_{m}\left\{\frac{g_{km}^{2}}{2\omega_{k}\omega_{m}}{\left(\omega_{k}^{2}-\omega_{m}^{2}\right)}^{2}ℜ\left[S\left(\omega_{k}+\omega_{m}\right)-S\left(\omega_{k}-\omega_{m}\right)\right]\right\},$$

and

(87) $$\begin{array}{ccc} \rho_{mk} & = & -\frac{g_{km}^{2}}{2\omega_{k}^{2}}[\left(\omega_{m}^{2}-2\omega_{m}\omega_{k}-\omega_{k}^{2}\right){\left(\omega_{m}-\omega_{k}\right)}^{2}ℜ\left[S\left(\omega_{m}-\omega_{k}\right)\right]\\ & & +\left(\omega_{m}^{2}+2\omega_{m}\omega_{k}-\omega_{k}^{2}\right){\left(\omega_{m}+\omega_{k}\right)}^{2}ℜ\left[S\left(\omega_{m}+\omega_{k}\right)\right]]\;. \end{array}$$

This is a set of coupled differential equations for the functions $T_{k}^{\left(n\right)}\left(\tau \right)$, that must be solved with the initial condition $T_{k}^{\left(n\right)}\left(0\right)=\delta_{nk}/\left(2\omega_{k}\right)$. From the solution, one could compute the total number of created particles, taking into account that, for t>T,

(88) $$\sum_{k}2\omega_{k}T_{k}^{\left(n\right)}=1+2\sum_{k}\langle |\beta_{nk}{|}^{2}\rangle \;,$$

and that the mean value of the number of created particles 〈N〉 is

(89) $$\langle N\rangle =\sum_{n}\langle N_{n}\rangle =\sum_{nk}\langle |\beta_{nk}{|}^{2}\rangle \;.$$

It is worth remarking that, while in the deterministic case, the mode coupling depends on the spectrum of the cavity [19], and for a random motion it occurs generically.

The solutions of Equation (85) will be described in a forthcoming work, as well as the computation of the spectrum of created particles.

## 6. Remarks on Cosmological Stochastic Particle Creation

As already mentioned in the Introduction, SPC has also been considered in the context of quantum field theory in curved spacetime, and in particular, in a cosmological scenario. Aassuming a flat Robertson–Walker metric

(90) $$ds^{2}=a^{2}\left(\eta \right)\left(-d\eta^{2}+dx^{2}\right)\;,$$

where η is the conformal time and a(η) is the scale factor of the Universe. The dynamical equation for the Fourier mode of momentum *k* of a free quantum scalar field ϕ is [9]

(91) $$\phi_{k}^{\prime \prime }+\left[k^{2}+m^{2}a^{2}+\left(\chi -1/6\right)Ra^{2}\right]\phi_{k}=0\;.$$

Here, a prime denotes a derivative with respect to the conformal time, *m* is the mass of the field, *R* the scalar curvature, and χ the coupling to the curvature. The metric may have a stochastic component, as described in Ref. [11].

If the quantum field ϕ is coupled with another field φ through $L_{int}=-\left(\lambda /2\right)\phi^{2}\varphi^{2}$, the equation for the $\phi_{k}$ modes reads

(92) $$\phi_{k}^{\prime \prime }+\left[k^{2}+m^{2}a^{2}+\left(\chi -1/6\right)Ra^{2}+\lambda a^{2}\langle \varphi^{2}\rangle \left(t\right)+\lambda a^{2}\xi_{\varphi }\left(t\right)\right]\phi_{k}=0\;.$$

This Langevin equation can be obtained formally by integrating out the field φ in the context of effective field theories [25]. The equation contains a dissipative term proportional to $\langle \varphi^{2}\rangle$ and a multiplicative noise $\xi_{\varphi }$ that describes the fluctuations of $\varphi^{2}$ around its mean value. Note that the equations for the modes correspond to a set of uncoupled harmonic oscillators with a time-dependent and stochastic frequency.

From the discussion above we see that there are several sources of stochastic behavior that may induce SPC for the quantum field. If the time scale of the stochastic fluctuations is much shorter than $H^{-1}$ (*H* is the Hubble constant), the time dependence of the nonstochastic component of the metric can be neglected, and the equations for the Fourier modes are of the form

(93) $$\phi_{k}^{\prime \prime }+\left[k^{2}+M^{2}\left(1+\epsilon \xi \left(t\right)\right)\right]\phi_{k}=0\;,$$

where *M* is a constant. We included a factor ϵ to make contact with our previous notation. This equation has been analyzed in Refs. [12,13], using the analogy with the time-independent one-dimensional Schroedinger equation

(94) $$-\frac{1}{2m}\frac{d^{2}\psi }{dx^{2}}+V_{r}\left(x\right)\psi =E\psi ,$$

for the wave function ψ(x) of an electron inside a wire with random impurities, modeled by a random potential $V_{r}\left(x\right)$.

It is usual to describe $V_{r}\left(x\right)$ as a set of potential barriers separated by a random distance, each barrier corresponding to a scatterer in the wire. Using the formalism of the transfer matrix, one can compute the reflection and transmission coefficients across a large number of scatterers, with the result [12]

(95) $$T=e^{-\frac{L\gamma }{\Delta x}}\;,$$

where *L* is the length of the wire, Δx the mean distance between scatterers, and γ a constant that depends on the shape of the barriers, the “Lyapunov exponent”. This the the phenomenon known as Anderson localization. Using the analogy between Equations (93) and (94), one can show that the random time-dependent mass in Equation (93) produces an exponential SPC, with

(96) $$\langle |\beta_{k}{|}^{2}\rangle \propto e^{\mu (k)\eta }\;,$$

where μ(k) is the *k*-dependent rate [12,13].

The results we found in Section 4 can be applied mutatis mutandis to the cosmological problem. In particular, instead of computing the SPC rate through the reflection and transmission coefficients, one can compute the stochastic mean value $\langle |\phi_{k}{|}^{2}\rangle$ using the stochastic version of multiple scale analysis. The result for the Bogoliubov coefficient $\beta_{k}$ associated with Equation (93) is

(97) $$\langle |\beta_{k}{|}^{2}\rangle =\frac{1}{2}\left(e^{\left(k^{2}+M^{2}\right)ℜ\left[S\left(2\sqrt{k^{2}+M^{2}}\right)\right]\epsilon^{2}\eta }-1\right)\;.$$

This is an alternative approach to this problem, which highlights the relation between the SPC rate and the correlation function of the noise. Moreover, the stochastic MSA could be generalized to more complex situations where, in addition to noise, there is a deterministic time dependence in the effective mass of the Fourier modes. This is the case, for instance, when the time dependence of the metric cannot be neglected, or when considering particle creation during reheating in the presence of quantum noise [26].

## 7. Conclusions

In this paper, we analyzed the phenomenon of particle creation produced by stochastic external conditions. We focused mainly on the DCE, for the case of an electromagnetic cavity in which one of the mirrors has a stochastic motion. Physically, the motion of the mirror can be produced by thermal fluctuations, or due to the coupling to another system (environment). We have presented both perturbative and nonperturbative evaluations of the number of created particles. The perturbative approach is valid for short times due to the presence of secular terms. To analyze the evolution of the system for longer times, we adapted the MSA to the stochastic motion of the mirror. Although the fact that the stochastic terms can produce parametric resonance for a single harmonic oscillator with multiplicative noise is well known [22], we have rederived those results using the conventional approach of MSA, and generalized the result to compute the Bogoliubov coefficients. We have also described the stochastic parametric resonance for the case of coupled harmonic oscillators. This is crucial for the DCE since, in general, a noisy moving mirror will couple the different electromagnetic modes in the cavity. Unlike the deterministic case, where the mode coupling takes place only when the spectrum of the cavity is such that the external frequency Ω satisfies $\Omega =|\omega_{k}\pm \omega_{j}|$ for some particular eigenfrequencies, the intermode coupling in the stochastic counterpart is generic. This is indeed due to the fact that the random motion of the mirror can be thought of as containing several frequencies.

Although the number of created particles is, in general, much smaller in the stochastic than in the deterministic situation, one could, in principle, observe this kind of phenomena in superconducting cavities. The deterministic DCE was first observed using a transmission line terminated by a SQUID, by applying a time-dependent magnetic flux through it [27]. The SPC could be observed in this context by applying a noisy magnetic flux. The spectrum of created particles would be very different in both cases, in particular, if the experiment is performed in a closed cavity.

We have also made contact with calculations of stochastic particle creation in a cosmological context. In this case, the different modes of the field are not coupled. Previous works in the subject emphasized the connection with the resistance in one-dimensional chains with impurities. Here, we have shown that the same results can be interpreted as produced by stochastic parametric resonance.

## Data Availability

Not applicable.
